# Impact of three field-practical thawing methods on bovine semen motility, viability, and membrane and acrosome integrity at 0 and 2 hours post-thaw

**DOI:** 10.29374/2527-2179.bjvm012025

**Published:** 2026-08-06

**Authors:** Alfredo Pozo-Curo, Dionet Keny Bellido-Quispe, Katherine Milagros Tacza Paquiyauri, Rogelio Sobero Ballardo, Luis Arturo Rodríguez Zamora, Mijail Contreras Huamani, Curtis R. Youngs

**Affiliations:** 1 Escuela Profesional de Medicina Veterinaria, Universidad Nacional de San Cristóbal de Huamanga, Ayacucho, Peru; 2 Laboratorio de Biotecnología Reproductiva, Estación Experimental Agraria Canaán, Instituto Nacional de Innovación Agraria, Ayacucho, Peru; 3 Department of Animal Science, Iowa State University, Ames, Iowa, United States

**Keywords:** bull semen, cryopreservation, spermatozoa, sperm quality, sperm damage, sêmen bovino, criopreservação, espermatozoides, qualidade espermática, danos espermáticos

## Abstract

The effectiveness of artificial insemination with frozen semen depends largely on semen quality, which can be influenced by the thawing method. This study evaluated the effect of three field-practical semen thawing methods on bovine sperm motility, viability, and plasma membrane and acrosome integrity at 0 and 2h post-thaw. The treatments were: T1 (water at 37 °C), T2 (axilla at 36.1°C), and T3 (room temperature at 23.4 °C). A total of 42 frozen semen straws was analyzed. Motility was assessed using a CASA system, viability was evaluated with Hoechst/propidium iodide staining, and plasma membrane and acrosome integrity were examined using the hypoosmotic swelling (HOS) test and Coomassie blue (CB) staining, respectively. Statistical analysis included ANOVA and t-tests. At 0h, thawing using a water bath (T1) or the axilla (T2) resulted in higher (P < 0.05) total motility, progressive motility, rapid motility, and number of rapidly motile sperm compared to thawing at room temperature (T3). Thawing using a water bath also resulted in higher (P<0.05) sperm viability and percentage of HOS+/CB+ cells at 0h than thawing with T2 or T3. At 2h, post-thaw, T1 resulted in superior (P<0.05) total motility, progressive motility, rapid motility, and viability compared with T2 and T3 thawing methods. Holding samples from 0 to 2h reduced sperm quality parameters in all three thawing methods. In conclusion, thawing semen in warm water at 37 °C yielded better sperm quality parameters at both 0 and 2h post-thaw than thawing in the axilla or at room temperature.

## Resumo

A eficácia da inseminação artificial com sêmen congelado depende, em grande parte, da qualidade do sêmen, que pode ser influenciada pelo método de descongelamento. Este estudo avaliou o efeito de três métodos de descongelamento de sêmen aplicáveis em condições de campo sobre a motilidade, a viabilidade e a integridade da membrana plasmática e do acrossoma de espermatozoides bovinos imediatamente após o descongelamento (0 h) e após 2 h. Os tratamentos foram: T1 (água a 37 °C), T2 (axila a 36,1 °C) e T3 (temperatura ambiente a 23,4 °C). Foram analisadas 42 palhetas de sêmen congelado. A motilidade foi avaliada por meio de um sistema de análise computadorizada de sêmen (CASA), a viabilidade foi determinada por coloração com Hoechst/iodeto de propídio, e a integridade da membrana plasmática e do acrossoma foi examinada pelos testes de inchaço hiposmótico (HOS) e coloração por azul de Coomassie (CB), respectivamente. A análise estatística incluiu ANOVA e teste t. No momento 0 h, o descongelamento em banho-maria (T1) ou na axila (T2) resultou em maiores valores (P < 0,05) de motilidade total, motilidade progressiva, motilidade rápida e número de espermatozoides com motilidade rápida em comparação ao descongelamento à temperatura ambiente (T3). O descongelamento em banho-maria também proporcionou maior viabilidade espermática (P < 0,05) e maior porcentagem de células HOS+/CB+ em 0 h, quando comparado aos métodos T2 e T3. Após 2 h do descongelamento, o tratamento T1 apresentou motilidade total, motilidade progressiva, motilidade rápida e viabilidade superiores (P < 0,05) em relação aos métodos T2 e T3. A manutenção das amostras entre 0 e 2 h reduziu os parâmetros de qualidade espermática em todos os métodos de descongelamento. Conclui-se que o descongelamento do sêmen em água aquecida a 37 °C proporciona melhores parâmetros de qualidade espermática tanto imediatamente após o descongelamento quanto após 2 h, em comparação com o descongelamento na axila ou à temperatura ambiente.

## Introduction

Artificial insemination (AI) is a widely employed strategy in bovine reproduction due to its direct impact on genetic improvement of cattle herds (Chakravarthi & Balaji, 2010; Patel et al., 2017). Implementation of this technique requires the selection of semen from sires with superior genetic traits and subsequent insemination of females, with the goal of increasing the quality and productivity of progeny (Zhang et al., 2024). Although cryopreserved semen is used extensively in various AI programs due to its advantages for global distribution, concern exists due to the potential for a low fertilization rate (Crespilho et al., 2014; Porras-Vargas et al., 2024).

The quality of cryopreserved semen for routine use in AI varies not only due to the freezing process but also because of the thawing method (Sharafi et al., 2022). During the freezing and thawing processes, sperm undergo structural and functional damage, collectively known as cryoinjuries. Factors such as the type of semen extender used (Crespilho et al., 2014), individual bull, breed (Ntemka et al., 2016; Solís et al., 2024), sperm concentration per straw, straw size (0.25 or 0.5 ml; Diskin, 2018), and protocols for freezing and thawing (Rajbari et al., 2022) can significantly influence post-thaw sperm quality and affect its fertilizing capacity.

Likewise, along with the aforementioned factors, the storage time after thawing may negatively affect sperm (Nguyen et al., 2023; Solís et al., 2024). These factors induce alterations in several key parameters of sperm quality, such as membrane integrity and fluidity, metabolic and enzymatic functions, and the induction of oxidative stress through the generation of free radicals (Crespilho et al., 2014; Ntemka et al., 2016; Solís et al., 2024). The oxidative stress causes cytoskeletal destabilization, ionic imbalances, macromolecular alterations, DNA fragmentation and epigenetic modifications, which negatively affect both spermatozoa quality parameters and fertilizing potential (Pini et al., 2018; Yánez-Ortiz et al., 2022).

Among the factors that can affect sperm quality, freezing and thawing techniques are likely the most important (Sharafi et al., 2022). Particularly, the methods used for thawing may be critical, as they directly influence sperm motility and functionality. In this regard, it typically is recommended to thaw frozen bovine semen straws at a physiological temperature. This process consists of immersing the straws in a water bath at 37 °C for 15 to 45 seconds (Rajbari et al., 2022).

Alternative methods, such as axillary thawing, have also been employed for semen thawing (Villa-Duque et al., 2016). This technique is useful due to its practicality under field conditions, especially when the necessary equipment for semen thawing is unavailable. However, it exhibits a significantly lower thermal transfer speed due to the low thermal conductivity of air and human tissues (Yamaji et al., 2013), which may result in slower and uneven thawing, thus affecting the structural and functional integrity of the sperm. Despite this, the acrosomal and plasma membrane integrity of spermatozoa obtained through this technique can be comparable to that observed with recommended methods (Villa-Duque et al., 2016).

Livestock rearing in Ayacucho, Perú occurs at elevations above 3500 meters, characterized by cold temperatures and extreme climate variations. Some inseminators employ ambient temperature thawing, as previously suggested (Rajbari et al., 2022). However, slow thawing may promote the recrystallization of intracellular content, which could damage cellular organelles (Nguyen et al., 2023). Previous studies have shown that lower thawing temperatures (e.g., 5°, 20°, or 24 °C) negatively impact post-thaw sperm motility (Aksu et al., 2024; Barth & Bowman, 1988; Rajbari et al., 2022). In fact, both sperm motility and the proportion of live sperm are adversely affected at lower thawing temperatures. This effect may be related to prolonged exposure to thermal shock and the recrystallization process, which significantly increases DNA damage in sperm (Aksu et al., 2024).

The paucity of research and the low consistency among published results hinder the establishment of clear recommendations regarding the optimal field-practical bovine semen thawing method. This uncertainty highlights the need to generate evidence that allows for the comparison of different protocols under controlled conditions. Therefore, the objective of the present study was to investigate the effects of thawing frozen bovine semen using warmed water (37 °C), the axillary body region (36.1 °C), or room temperature (23.4 °C) on sperm motility, viability, and plasma membrane and acrosome integrity parameters at 0h and 2h post-thaw.

## Methods

### Workplace and origin of semen

The experiment was conducted at the Laboratorio de Biotecnología Reproductiva of the Estación Experimental Agraria Canaán of the Instituto Nacional de Innovación Agraria (INIA) and the Laboratorio de Biotecnología y Reproducción of the Escuela de Medicina Veterinaria at the National University of San Cristóbal de Huamanga.

A total of 45 straws of bovine semen cryopreserved in 0.25 ml straws at a concentration of 30 x 10^6^ sperm/straw was provided for this study by PROMEG (Proyecto Nacional de Mejoramiento Genético) personnel**.** Straws originated from a single bull from each of three different breeds (Angus, Braunvieh, Simmental; 15 straws per bull). Different breeds were specifically chosen in an attempt to capture the biological variation that exists among AI bulls, with the intent that results will be robust.

### Experimental design, semen thawing and sperm analysis

The experimental design used was a randomized complete block design, with breed as the block to ensure that any potential effect of breed would be stratified across all three thawing treatments.

Cryopreserved straws were randomly assigned within breed to one of three different thawing protocols, all of which were conducted by the same person to reduce variation due to technician. For thawing protocol 1 (T1), individual semen straws were removed from the LN_2_ tank and immediately placed into a thermos containing water preheated to 37°C. The straw remained fully submerged for 45 seconds to ensure rapid and homogeneous thawing. For thawing protocol 2 (T2), individual semen straws were placed in the axillary region of the same technician for 45 seconds, with axillary temperature being measured with a digital thermometer (36.1 ± 0.2°C, mean ± SEM). For thawing protocol 3 (T3), straws were removed from the LN2 tank and placed on a clean, dry surface at room temperature (23.4 ± 0.5°C) for 45 seconds. After thawing, the contents of the straws for all treatments were transferred to a 1.5 ml Eppendorf tube pre-warmed to 37°C.

Sperm motility, viability, and plasma and acrosomal membrane integrity were evaluated at 0h (immediately after thawing) and 2h post-thaw. During this period, all semen samples were maintained at 37°C on a heated stage (Minitüb GmbH, Tiefenbach, Germany).

### Sperm motility analyses using CASA

Sperm motility analyses were performed using a computer-assisted semen analysis (CASA) system (AndroVision®; Minitüb GmbH, Tiefenbach, Germany) using the bovine semen settings. Sperm were visualized using a phase-contrast microscope at 20X magnification (Axiolab, Carl Zeiss, Oberkochen, Germany). For each sample, 3 μL of semen were placed into a 20 μm height Leja® counting chamber (Minitüb GmbH, Tiefenbach, Germany) on a stage heated to 37°C. All analyses were conducted according to manufacturer’s instructions, reading three different fields and recording the average reading for the final result.

In addition, the use of threshold settings in CASA allowed for the classification of spermatozoa into subpopulations. The playback function was used to visually control the limits according to the visual motility trajectories. Five subgroups were defined as following: a) rapid motility (Velocity Curved Line [VCL] >50 µm/s and straightness of the mean path [STR] >70), b) slow motility (VCL<80 µm/s), c) circular motility (coefficient of sperm movement-ratio >10 µm/s and radial coefficient of sperm movement ratio <80 µm/s and rotation >0.70), d) local motility (VCL >10 µm/s and Velocity Straight Line [VSL] <48 µm/s), and e) immotile (VCL <24 µm/s and amplitude of lateral head displacement [ALH] <1.00)

### Sperm viability analysis

Sperm viability was evaluated using the Hoechst 33342/propidium iodide (PI) viability kit (Hoechst 33342/PI, Minitüb GmbH, Ref. 15407/0009, Lot. 90400429704) according to the manufacturer's instructions. Briefly, 50 μl of thawed semen were incubated with 1.5 μl of stain for 15 minutes at 37 °C, avoiding exposure to light. After incubation, sperm viability was assessed using the aforementioned CASA/microscope system equipped with fluorescence microscopy. A minimum of 200 sperm were analyzed per sample.

### Plasma membrane and acrosome integrity analyses

Evaluation of plasma membrane and acrosomal integrity was performed according to a previously published method (Carretero et al., 2019) with minor modifications. Briefly, 12.5 µl of semen were incubated at 37°C for 20 min in 50 µl of a hypoosmotic (50 mOsm) fructose-sodium citrate solution. Samples were subsequently fixed with 62.5 µl of 4% paraformaldehyde in phosphate-buffered saline (PBS) for 4 min at room temperature. Thereafter, samples were centrifuged at 800 g for 10 min, and the sperm pellet was re-suspended in 100 µl of PBS. A 50 µl drop of the suspension was evenly dispersed on a microscope slide and allowed to air dry. Finally, samples were stained with 0.22% Coomassie Blue (CB) for 5 min prior to evaluation at 100x magnification using an immersion oil lens on a brightfield microscope (AmScope B120). A total of 200 sperm was evaluated and classified into one of four categories: (1) sperm with a functional plasma membrane (HOS+) and presence of acrosome (CB+), (2) sperm with a functional plasma membrane (HOS+) and absence of acrosome (CB-), (3) sperm with a non-functional plasma membrane (HOS-) and presence of acrosome (CB+), and (4) sperm with a non-functional plasma membrane (HOS-) and absence of acrosome (CB-).

### Statistical analysis

All data analyses were performed using SPSS® Statistical Software package v.26 (IBM®, Armonk, New York, USA). Analysis of variance (ANOVA) was used to compare thawing methods, provided that the assumptions of normality and homoscedasticity were met; otherwise, the Kruskal-Wallis test was applied. To analyze differences between 0h and 2h time points, the Student’s t-test related samples test was used when the assumption of normality was fulfilled; otherwise, the non-parametric Wilcoxon test was used. Comparisons between treatments were conducted using Tukey's test or the Games-Howell test, as appropriate. Differences were considered statistically significant with a Type I error rate of less than 5% (P < 0.05). Results are presented as mean ± standard error of the mean (SEM).

The study complied with the ethical principles of scientific research (CONSTANCIA N° 004-2025/CEI-VRI-UNSCH - AYACUCHO). The experimental protocol was submitted for evaluation and approved by the Ethics Committee of the National University of San Cristóbal de Huamanga. Although the study does not involve interventions with live animals, the traceability of biological material and the responsible handling of samples were ensured in accordance with bioethical standards and good laboratory practices.

## Results

### Effects on sperm motility

At time 0h, total motility and progressive motility were higher (P<0.05) in samples thawed using T1 and T2 methods, compared with T3. However, at 2h post-thaw, samples from T1 showed higher (P<0.05) total and progressive motility than samples thawed using methods T2 or T3. Additionally, in all treatments, total and progressive motility decreased significantly at 2h compared to 0h (Table 1).

**Table 1:** Total and progressive sperm motility at 0h and 2h post-thaw of cryopreserved bovine semen thawed using different methods.

**Semen thawing method**	**n**	**Total motility (%)**		**Progressive motility (%)**	
		**0 hours**	**2 hours**	**0 hours**	**2 hours**
T1	15	78.0±2.3^a,A^	68.2±3.2^a,B^	69.8±2.8^a,A^	59.5±3.4^a,B^
T2	15	69.4±2.8^a,A^	50.7±4.7^b,B^	59.8±2.6^a,A^	42.4±4.1^b,B^
T3	12	46.9±6.0^b,A^	41.1±6.3^b,B^	37.8± 4.7^b,A^	33.5±5.0^b,B^

T1: Warm water at 37°C for 45 sec; T2: Axillary temperature at 36.1 ± 0.2°C for 45 sec; T3: Room temperature at 23.4 ± 0.5°C for 45 sec. n: number of straws. Results are presented as mean ± standard error of the mean (SEM). ^a,b^ Unlike lowercase letters within the same column indicate statistical differences (P<0.05). ^A,B^ Unlike uppercase letters within the same row for the same variable indicate statistical differences (P<0.05). During the experiment, three straws in T3 were lost due to biological manufacturing defects (0 Angus, 1 Braunvieh, and 2 Simmental).

At 0h post-thaw, rapid motility was higher (P<0.05) in treatments T1 and T2 than in T3, whereas no differences (P>0.05) were observed in slow, circular, and local motility among the three treatments. At 2h post-thaw, rapid motility was higher (P<0.05) for samples thawed using method T1 than method T2 or T3. No differences (P>0.05) in slow, circular, or local motility were observed among thawing methods T1, T2, and T3 (Table 2).

**Table 2:** Rapid, slow, circular, and local sperm motility at 0h and 2h post-thaw of cryopreserved bovine semen thawed using different methods.

**Semen thawing method**	**n**	**Type of sperm motility (%)**							
		**Rapid motility**		**Slow motility**		**Circular motility**		**Local motility**	
		**0 hours**	**2 hours**	**0 hours**	**2 hours**	**0 hours**	**2 hours**	**0 hours**	**2 hours**
T1	15	52.7±3.0^a,A^	47.0±3.0^a,B^	14.1±1.5^a,A^	12.7±1.7^a,A^	0.5±0.2^a,A^	0.2±0.08^a,B^	8.7±0.8^a,A^	8.8±0.9^a,A^
T2	15	45.3±2.0^a,A^	31.1±2.7^b,B^	14.0±1.7^a,A^	11.3±1.9^a,B^	0.4±0.2^a,A^	0.05±0.03^a,B^	9.6±0.6^a,A^	8.3±0.8^a,A^
T3	12	25.5±3.1^b,A^	23.9±3.2^b,A^	12.0±2.5^a,A^	9.5±2.3^a,B^	0.2±0.1^a,A^	0.1±0.05^a,A^	9.2±1.4^a,A^	7.6±1.4^a,A^

T1: Warm water at 37°C for 45 sec; T2: Axillary temperature at 36.1 ± 0.2°C for 45 sec; T3: Room temperature at 23.4 ± 0.5°C for 45 sec. n: number of straws. Results are presented as percentage mean ± standard error of the mean (SEM). ^a,b^ Unlike lowercase letters within the same column indicate statistical differences (p<0.05). ^A,B^ Unlike uppercase letters within the same row for the same variable indicate statistical differences (p<0.05).

Rapid and circular motility declined (P<0.05) between 0 and 2h in treatments T1 and T2, whereas no changes (P>0.05) in rapid and circular motility were observed in samples thawed using method T3. Slow motility decreased (P<0.05) between 0 and 2h post-thaw in samples thawed using methods T2 and T3, but not for samples thawed using method T1. There were no changes (P>0.05) in local motility between 0 and 2 hours among any of the thawing treatments (Table 2).

### Effects on sperm viability

**Figure 1:**
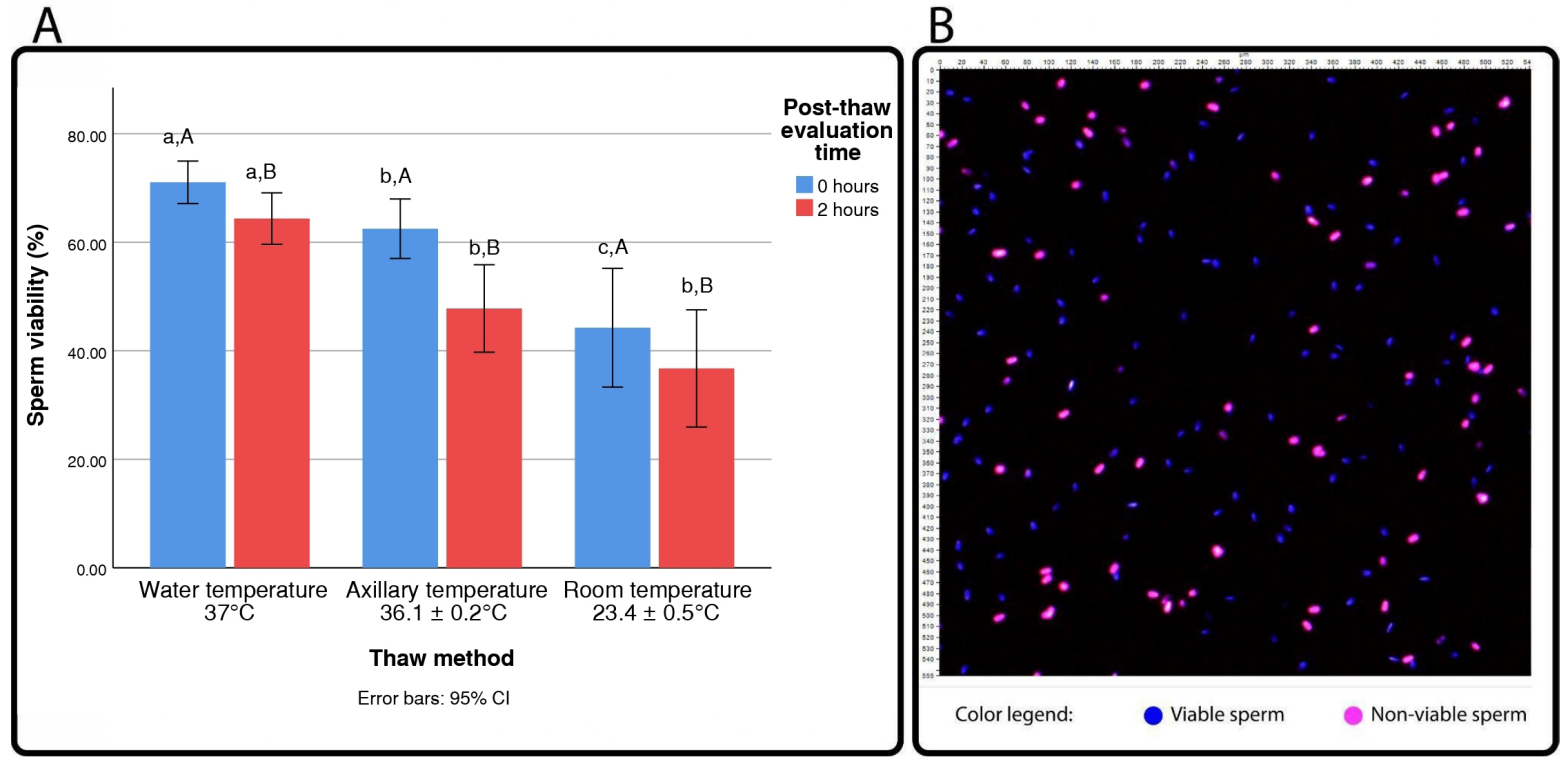
Viability of bull sperm assessed by Hoechst 33342/propidium iodide (PI) staining. (A) Sperm viability (%) at 0h and 2h after using different thawing methods: 37 °C water bath (T1), 36.1°C axillary region (T2), or 23.4°C room temperature (T3). ^a,b,c^ Unlike lowercase letters across thawing methods at 0 and 2 hours indicate statistical differences (P<0.05). ^A,B^ Unlike letters within a thawing method at 0 and 2 hours indicate a statistical difference (P<0.05); (B) Viable and non-viable sperm [[Q2: Q2]].

Sperm viability at 0h and 2h post-thaw was higher (P<0.05) in T1 compared with T2 or T3 (Figure 1A). Concerning the effect of time, viability decreased (P<0.05) in all treatments at 2 hours compared with 0 hours (Figure 1A). Fluorescence indicating cell viability can be seen in Figure 1B.

### Effect on plasma membrane and acrosome integrity

**Figure 2:**
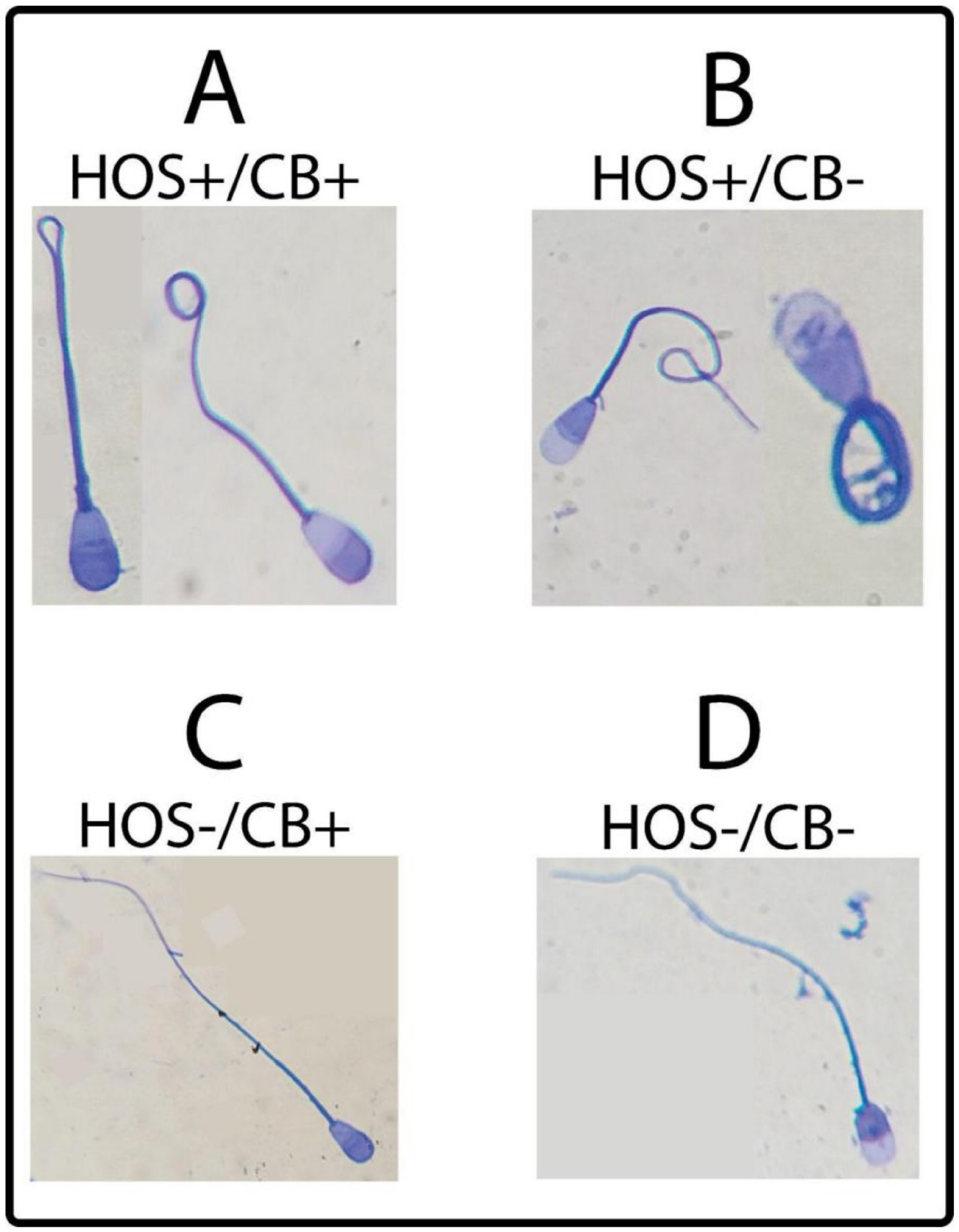
Functionality of the plasma membrane and acrosome integrity of frozen-thawed bovine sperm. (A) Sperm with a functional plasma membrane and presence of acrosome (HOS+/CB+); (B) Sperm with a functional plasma membrane and absence of acrosome (HOS+/CB-); (C) Sperm with a non-functional plasma membrane and presence of acrosome (HOS-/CB+); (D) Sperm with a non-functional plasma membrane and absence of acrosome (HOS-/CB-).

At 0 hour, T1 had a superior (P<0.05) functional plasma membrane and intact acrosome (HOS+ and CB+) compared to T2 and T3. At 2 hours, T1 continued to be superior to T3. When comparing the values between 0 and 2h, a decline (P<0.05) was observed in T1 and T3, in contrast to T2, which did not differ (P>0.05; Table 3 and Figure 2A).

**Table 3:** Sperm plasma membrane and acrosome integrity at 0h and 2h post-thaw of cryopreserved bovine semen thawed using different methods.

**Semen thawing method**	**n**	**Plasma membrane and acrosome integrity (%)**							
		**HOS+/CB+**		**HOS+/CB-**		**HOS-/CB+**		**HOS-/CB-**	
		**0 hours**	**2 hours**	**0 hours**	**2 hours**	**0 hours**	**2 hours**	**0 hours**	**2 hours**
T1	15	55.3±2.6^a,A^	50.2±2.5^a,B^	1.1±0.3^a,A^	1.9±0.5^a,B^	41.1±2.8^b,A^	45.1±2.7^b,B^	2.4±0.5^a,A^	2.7±0.5^a,,A^
T2	12	46.1±2.1^b,A^	43.6±2.0^a,b,A^	0.7±0.2^a,A^	1.3±0.3^a,B^	50.8±2.3^a,A^	52.7±2.3^a,b,A^	2.4±0.4^a,A^	2.5±0.4^a,A^
T3	12	45.1±2.7^b,A^	38.4±2.8^b,B^	1.2±0.3^a,A^	2.2±0.4^a,B^	50.4±2.9^a,b,A^	55.6±3.1^a,B^	3.3±0.7^a,A^	3.9±0.4^a,A^

T1: Warm water bath at 37°C for 45 sec, T2: Axillary temperature of 36.1 ± 0.2°C for 45 sec, T3: Room temperature of 23.4 ± 0.5°C for 45 sec. n: number of straws. HOS+: spermatozoa with a functional plasma membrane. HOS-: spermatozoa with a non-functional plasma membrane. CB+: presence of acrosome. CB-: absence of acrosome. Results are presented as means ± standard error of the mean (SEM). ^a,b^ Unlike lowercase letters in the same column indicate statistical differences (p < 0.05). ^A,B^ Unlike uppercase letters in the same row for the same variable indicate statistical differences (p < 0.05). Three samples in T2 were excluded from the analysis due to technical failures during slide preparation, which resulted in the loss of biological material.

For HOS+ and CB− sperm, no differences (P>0.05) were observed between treatments at either 0 or 2h. However, all treatments showed a decline (P<0.05) in the proportion of sperm with plasma membrane and acrosome integrity (Table 3 and Figure 2B).

In the case of HOS− and CB+ sperm, T1 showed less damage (P<0.05) than T2 at 0 h and T3 at 2 h. In addition, differences (P<0.05) were observed between 0 and 2h in T1 and T3, whereas no significant changes were recorded in T2 between the two times (Table 3 and Figure 2C).

Finally, in HOS- and CB- spermatozoa, no differences were detected between treatments at 0h and 2h. Likewise, no significant differences were found between 0 and 2h in any of the treatments (Table 3 and Figure 2D).

## Discussion

Bovine AI technicians in our locale, which is characterized by high elevation (>3500 masl) and cold nighttime temperatures (<0°C), often utilize ambient temperature thawing due to lack of a temperature-regulated thawing device. This lack of proper thawing devices exists in other parts of the world. This study was conducted to assess, under controlled conditions, the impact of three different field-practical thawing methods, which included a thawing temperature lower than that typically recommended.

Results of our study demonstrated that the percentage of total motility, progressive motility, and rapid motility were higher at 0h when thawing bovine semen in the warm water bath (T1: 37°C) or axillary region (T2: 36.1°C) compared to thawing at room temperature (T3). Moreover, sperm viability and the percentage of HOS+/CB+ sperm were also higher at 0h when thawing in warm water. At 2h, thawing with warm water resulted in better values for several parameters—including total motility, progressive motility, rapid motility, and viability—compared to thawing in the axilla or at room temperature. In addition, maintaining samples from 0h to 2h negatively affected many parameters within each thawing method.

Cryopreserved semen samples may exhibit variations in sperm quality associated with several factors, particularly the freezing and thawing processes (Sharafi et al., 2022). Semen thawing represents a critical phase, as the conditions employed—specifically the thawing temperature and duration—can directly influence the quality of thawed spermatozoa, particularly sperm kinetics (Rajbari et al., 2022; Zenteno et al., 2022). Higher total and progressive motility values have been reported when thawing is performed at 37 °C (Rajbari et al., 2022), a result consistent with our findings. This protocol, which involves thawing semen at 37 °C for 30 seconds, is also a widely adopted practice in the field (Nur et al., 2003; Rastegarnia et al., 2013). Indeed, some previous studies suggest that a thawing temperature between 35 and 37°C promotes a more efficient metabolic reactivation of frozen sperm, resulting in superior motility. Lower temperatures (e.g., 5°, 20°, or 24 °C) negatively affect sperm motility (Aksu et al., 2024; Barth & Bowman, 1988; Rajbari et al., 2022). In fact, both sperm motility and the proportion of live sperm cells are compromised at lower thawing temperatures. This effect may be related to prolonged exposure to thermal shock and the recrystallization process, which significantly increases sperm DNA damage (Aksu et al., 2024).

On the other hand, thawing semen at very high temperatures for a shorter duration may be more beneficial compared to 37 °C. Thawing bull semen at 65-70 °C for 7 sec significantly increased post-thaw sperm motility and viability in one study (Lyashenko, 2015). In a study with buffalo semen (Rastegarnia et al., 2013), initial post-thaw sperm motility was higher when a higher temperature and shorter duration (70 °C for 6 sec) were used compared to the lower standard temperature (37 °C for 30 seconds). In a more recent study (Aksu et al., 2024), thawing at 38 °C for 30 seconds gave a result comparable to that obtained with thawing at 70 °C for 7 sec. Higher thawing temperatures for shorter duration reduce the harmful effects associated with recrystallization and rehydration processes (Lyashenko, 2015; Rajbari et al., 2022; Rastegarnia et al., 2013).

The T2 and T3 thawing treatments resulted in low post-thaw sperm motility rates. Human tissues and air are known to have a low rate of heat transfer, that is, low thermal conductivity (Yamaji et al., 2013). Therefore, hot water likely exhibits higher thermal conductivity for thawing straws of semen, which is reflected in higher post-thaw sperm motility rates. In fact, several studies have evaluated air-thawing methods, such as using the breast pocket (Barth & Bowman, 1988; Pickett & Berndtson, 1974), exposure to open air (Rajbari et al., 2022), or wrapping straws in paper towels and placing them on a thermal plate at 37°C for 3 minutes (DeJarnette & Marshall, 2005), and in all cases a reduction in sperm motility was observed. However, thawing straws using the axillary region or ambient temperature could represent practical options under field conditions for AI technicians.

Regarding storage time, our results showed that total motility and progressive motility declined in all treatments during the 2h period immediately post-thaw; moreover, rapid and circular motility also lessened during the first 2h post-thaw in T1 and T2. These findings are consistent with previous studies indicating that post-thaw sperm kinetics may be affected by storage time (Botta et al., 2019; Lyashenko, 2015; Ntemka et al., 2016). Furthermore, although the thawing treatments T1 and T2 did not show differences in total, progressive, or rapid motility at 0h, a decrease was observed at 2h. Approximately 25% of sperm became static after 3h of incubation at 36°C, demonstrating that higher temperatures cause more intense and earlier cellular exhaustion; a change in the sperm movement pattern is already observed during the first hour, with a reduction in cells exhibiting rapid movement. After the second hour, the rapidly moving cells became static (Botta et al., 2019), which is in agreement with our findings that showed a reduction in rapid motility at 2h. The increase in thermal energy may lead to a decrease in total motility, progressive motility, and sperm velocity in bovines (Rahman et al., 2014).

The present study also supports the findings of a previous study (Catena & Cabodevila, 1999) which demonstrated that sperm thermoresistance gradually decreases after thawing, with the most pronounced reduction in motility and vigor occurring after two hours of incubation. This underscores the importance of assessing post-thaw parameters at different time points, as functional stability is as critical as the initial semen quality.

Thawing bull sperm at 32°C showed viability comparable to 38 °C and 70 °C, whereas 24 °C resulted in reduced viability (Aksu et al, 2024). In our study, T1 showed significantly higher viability at 0h and 2h, consistent a previous report (Mehrez, 2001) that highlighted the importance of rapid and controlled thawing. The progressive decline in viability over time observed in all treatments confirms the degradative nature of the post-thaw process (Correa et al., 1996; Rastegarnia et al., 2013), with the extent of damage depending on the method used. Thawing at 37°C likely provided greater plasma membrane stability and osmotic recovery (Zenteno et al., 2022), while lower temperatures have been associated with thermal injury and reduced acrosomal integrity (DeJarnette et al., 2000). Interestingly, the optimal thawing range lies between 40 °C and 75 °C (Limin et al., 2014), where sperm structural integrity is better preserved. Overall, our results support that rapid thawing close to 37 °C is essential to preserve sperm structural and functional integrity after cryopreservation

The combined test for functional plasma membrane integrity (HOS+) and intact acrosome (CB+) showed that T1 treatment maintained a higher proportion of structurally viable sperm at both evaluation times than did T2 or T3. These results are in accordance with a previous observation (Nagy et al., 2004) that the initial stability of these structures is positively correlated with a lower rate of structural deterioration over time after thawing. Likewise, the progressive deterioration in T2 and especially in T3 highlights the risk of irreversible damage due to inadequate thawing (Rajbari et al., 2022; Zenteno et al., 2022), who contended that thawing at a moderate temperature is favorable for sperm preservation, as was performed in T1 treatment.

Additionally, lipids are an important constituent of the plasma membrane and confer a certain degree of fluidity to this structure, which depends, among other factors, on the temperature to which the cells are exposed (Kumar & Atreja, 2012). Cells exposed to oxidative stress may undergo lipid peroxidation, a phenomenon that also alters plasma membrane fluidity and impairs cellular transport (Gutteridge & Halliwell, 2010). The effects of lipid peroxidation on sperm function are mainly observed in reduced motility and altered plasma membrane fluidity (Aitken, 2017). Sperm plasma membrane damage increased over incubation time across all thermal treatments, with a higher incidence of damaged plasma membranes in samples incubated at the highest temperature (Botta et al., 2019).

## Conclusion

This study showed that thawing cryopreserved bovine semen in a 37°C water bath (T1) or 36.1°C axilla (T2) produced higher total, progressive and rapid motility, immediately post-thaw (0h) than thawing at room temperature (T3). Sperm viability and the proportion of HOS+/CB+ sperm were greater at 0 hours when thawed with T1. After 2h, thawing at T1 produced better results in terms of total, progressive, and rapid motility, and viability, compared to thawing methods at T2 or T3. Holding thawed bovine sperm for 2h adversely influenced several parameters, suggesting that the interval from thawing to insemination must be as brief as possible when using a field-practical thawing method such as in T2.
